# Kissing Aneurysms of the Anterior Communicating Artery Treated With Surgical Clipping: A Case Report and Literature Review

**DOI:** 10.7759/cureus.60824

**Published:** 2024-05-22

**Authors:** Corneliu Toader, Razvan-Adrian Covache-Busuioc, Bogdan-Gabriel Bratu, Antonio-Daniel Corlatescu, Andrei Adrian Popa, Alexandru Vladimir Ciurea

**Affiliations:** 1 Department of Neurosurgery, “Carol Davila” University of Medicine and Pharmacy, Bucharest, ROU; 2 Department of Neurosurgery, National Institute of Neurology and Neurovascular Diseases, Bucharest, ROU; 3 Department of Neurosurgery, Sanador Clinical Hospital, Bucharest, ROU

**Keywords:** conventional angiography, 3d angiography, coiling, surgical clipping, anterior communicating artery, kissing aneurysms

## Abstract

Intracranial “kissing” aneurysms are rare vascular conditions described as two anatomically adjacent aneurysms originating from either the same or different arteries, with their walls pressed together. Two-dimensional angiography was formerly considered the gold standard for diagnosis, with the three-dimensional rotational type now offering more insightful details about vascular discrepancies. The treatment of anterior communicating artery (AcoA) “kissing” aneurysms poses significant challenges, with surgical clipping proving difficult due to their deep midline location or the bilateral anterograde arterial supply. However, advancements in endovascular coil embolization, such as dual-volume reconstruction, can assist in diagnosis. This study presents the case of a 50-year-old patient who was diagnosed with “kissing” aneurysms of the AcoA. The patient underwent surgical clipping and showed no pathological follow-up findings. The surgical intervention often provides a more direct and effective approach. This case contributes to the body of knowledge surrounding the management of this complex disease.

## Introduction

Intracranial “kissing” aneurysms are a rare subtype of multiple aneurysms, characterized as two adjacent aneurysms that originate from either the same or different arteries, each having separate origins but partially adherent walls [1]. While the prevalence of intracranial aneurysms in the general population is estimated at about 3.2% [2], kissing aneurysms represent fewer than 1% of all intracranial aneurysms [1].

The unique proximity and shared walls of kissing aneurysms can lead to misdiagnosis as a single aneurysm, potentially resulting in inappropriate treatment and increasing the risk of subsequent rupture [3]. The most suitable imaging techniques for diagnosing kissing aneurysms are a subject of debate. While conventional angiography was previously considered the gold standard, three-dimensional (3D) rotational angiography now offers more detailed information about vascular anomalies, including the aneurysm’s origin artery, size, and direction [4].

Kissing aneurysms were first documented by Jefferson in 1978 [5]. Since then, numerous case reports and a few small series (≤5 cases) have been published [6,7]. Inci and Karakaya reported an incidence of 3.6% in their retrospective review of an aneurysm series, identifying 30 patients with kissing aneurysms among 1,073 aneurysms in 817 operated patients [8].

In 2021, Inci and Karakaya proposed a classification of kissing aneurysms which had a major impact on the clip selection as well as the evaluation of the intraoperative difficulties based on the spatial relationship of the aneurysms on the parent artery from the surgeon’s perspective. The classification is divided into the following three types: type I (proximal/distal) - one aneurysm is positioned proximally and the other distally on the parent artery (this classification is particularly relevant in scenarios where one aneurysm may obscure the approach to or complicate the treatment of the other, affecting the surgical strategy); type II (superior/inferior) - one aneurysm is located superiorly and the other inferiorly (this arrangement can influence the order of clipping, as accessibility to one might be impeded by the position of the other, necessitating strategic planning in the clipping sequence); type III (right/left) - one aneurysm is located to the right and the other to the left (this type may present challenges in terms of ensuring clear surgical access and avoiding compromise of the parent artery during clipping) [8].

These classifications are instrumental in selecting surgical clips and managing intraoperative challenges by systematizing the understanding of the spatial arrangement of kissing aneurysms. This structured approach enables surgeons to foresee and navigate potential obstacles concerning the visibility and access to aneurysms during microsurgery. For instance, being aware of the specific type of kissing aneurysms can guide surgeons in selecting the correct size, shape, and orientation of clips to effectively secure each aneurysm without endangering the parent artery or neighboring structures. Additionally, this understanding aids in strategizing the sequence of clipping, which is essential for reducing the risk of intraoperative rupture and ensuring a successful surgical outcome.

Recently, there has been controversy and complexity in the surgical management of kissing aneurysms, particularly regarding the positioning of clips. In some instances, only one of the aneurysms is clipped [9]. This article highlights current treatment approaches for these unique vascular conditions.

## Case presentation

A 50-year-old male patient was admitted to our clinic with cephalgia, dizziness, and a confusional state. At the neurological examination, the patient was conscious and confused, with elements of expressive aphasia, intracranial hypertension syndrome, and neck pain without neurological deficits.

**Figure 1:**
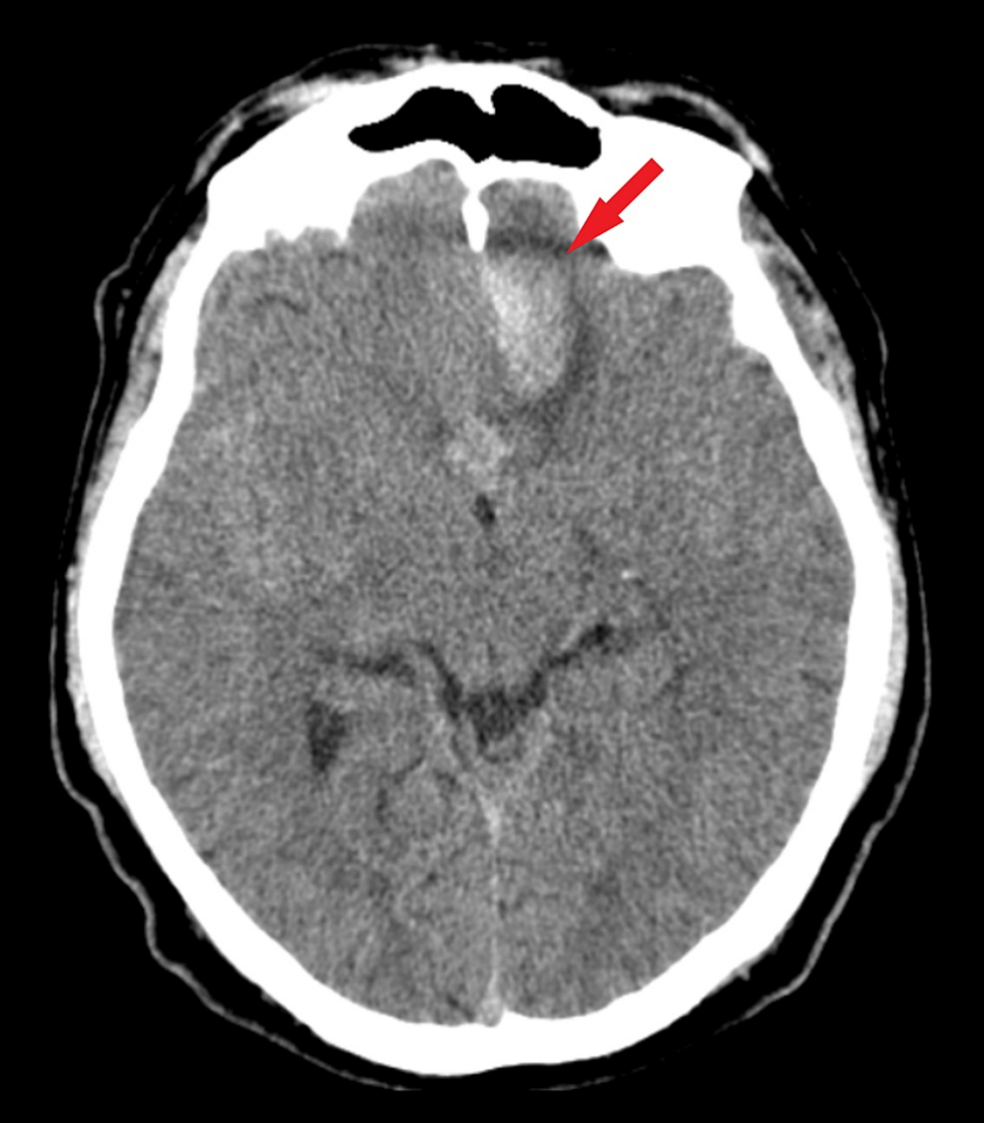
Preoperative CT scan. The axial section of the CT scan highlights a frontobasal hematoma with perilesional edema (red arrow).

**Figure 2:**
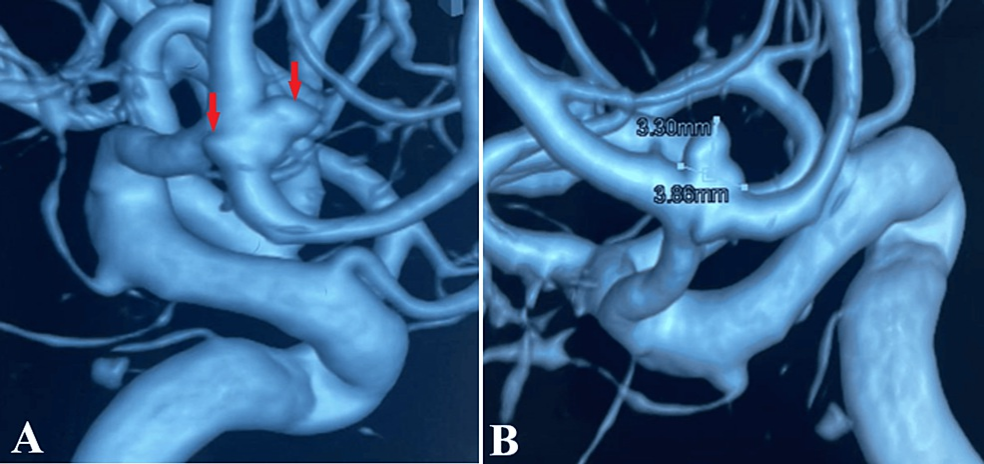
Preoperative three-dimensional digital subtraction angiography. Three-dimensional digital subtraction angiography was obtained after analyzing multiple angiography bidimensional images. Part A depicts both anterior communicating artery aneurysms (red arrow), while part B precisely measures the anterior aneurysm, with a median diameter of 3.3 mm and a neck size of 3.86 mm.

A CT scan revealed a subarachnoid hemorrhage (Fisher score 4) with left frontobasal hematoma (Figure 1). Therefore, two-dimensional (2D) and 3D digital subtraction angiography were done to confirm a possible vascular malformation. At the level of the anterior communicating artery (ACoA), an anteriorly ruptured saccular aneurysm was found, with a maximum diameter of 3.5 mm. Moreover, a second aneurysm posteriorly orientated with a maximum diameter of 2 mm was revealed, concluding the diagnosis of kissing aneurysms (Figure 2).

**Figure 3:**
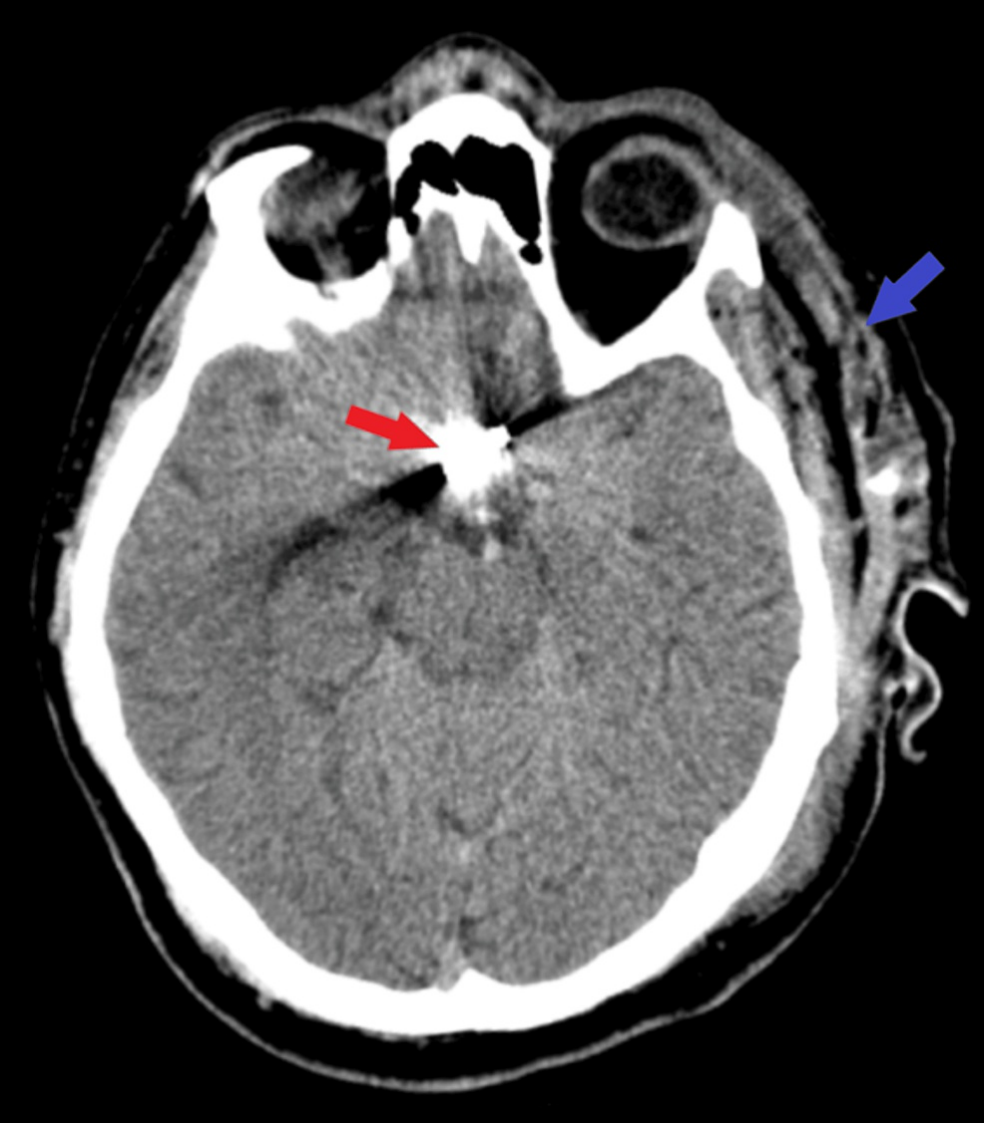
Postoperative CT scan. The axial section of tissue window CT examination demonstrates the proper clip positioning (red arrow), along with successful aneurysm clipping. A dehiscence lesion was observed on the left side (blue arrow) which was subsequently remitted.

Considering the patient’s state and the type of aneurysm, a microsurgical procedure was performed with a left parasagittal frontobasal flap, and successful clipping of the ACoA aneurysm was achieved, along with complete hematoma evacuation (Figure 3). Postoperative evolution was initially favorable, with a vasospasm episode being reported, causing degradation of the conscious state. After cerebral vasodilation therapy, anticoagulant, and triple H therapy, cerebral vasospasm was remitted. At discharge, the patient was conscious and coherent, with slightly remitted expressive aphasia and remitted intracranial hypertension syndrome.

**Figure 4:**
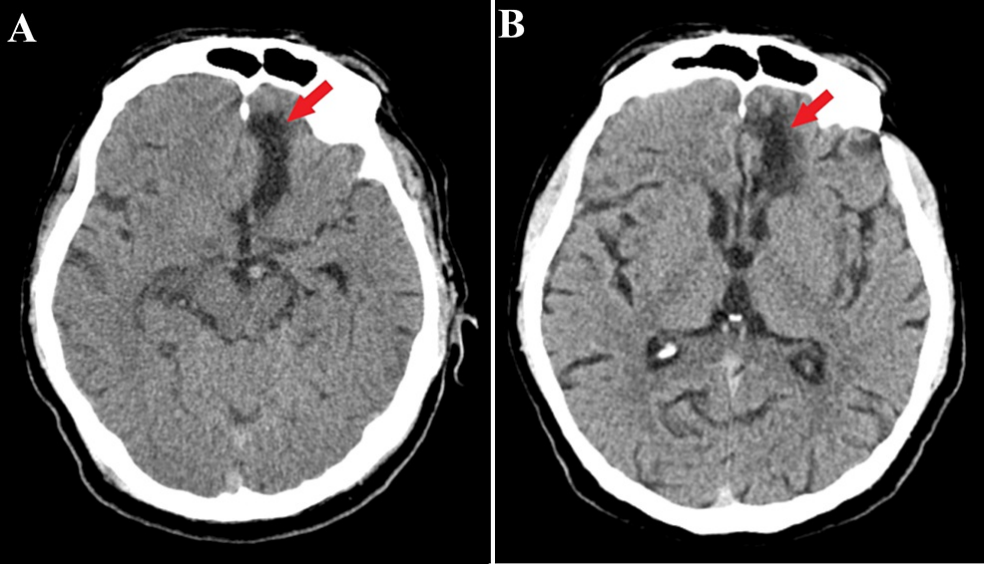
Follow-up CT scans. The axial sections of the CT scan at the four-month follow-up (A) and five-year follow-up (B) highlight a hypodensity area corresponding to the craniectomy (red arrows) and no indications of a recent hemorrhage.

During the four-month follow-up, the neurological state had improved, with mild cognitive disorders and right pyramidal syndrome. A CT examination highlighted the correct disposition of the cerebral clip with a right frontobasal hypodensity sequalae area and without signs of recent hemorrhage. Additionally, one-, two-, and five-year follow-ups revealed the exact clinico-radiological findings as the four-month follow-up (Figure 4). Notably, no other modifications were found and the patient received conservative treatment with antiepileptic and nootropics drugs.

## Discussion

Kissing aneurysms are characterized as two anatomically adjacent aneurysms originating from different arteries, with their walls partially pressed together [10]. The development of multiple aneurysms at the ACoA is influenced by various factors, including hemodynamic stress. This stress can be a contributing factor, particularly if the proximal anterior cerebral arteries (ACAs) are unequal in size, leading to aneurysm formation at the juncture of the ACoA and the A2 segment, often on the side of the dominant A1 segment [11]. Harada et al. categorized kissing aneurysms into two types based on the location of the aneurysmal neck. Type 1 includes aneurysms where each neck is on the same parent artery, whereas in Type 2, each arises from different parent arteries. Kissing ACoA aneurysms fall under Type 1 [12].

While CT angiography (CTA) and MR angiography are commonly used for the initial diagnosis of cerebral aneurysms and are helpful in identifying kissing aneurysms and determining the kissing and ruptured points, their application in evaluating aneurysms post-endovascular treatment (EVT) poses greater challenges [13].

Diagnosing multiple, kissing, or mirror ACoA aneurysms using conventional angiography can be difficult preoperatively. These multiple ACoA aneurysms are often misinterpreted as aneurysmal bleb, single multilobed, or bilobed saccular aneurysms [11]. Distinguishing between single multilobed and multiple saccular aneurysms is crucial for surgical planning and preventing intraoperative complications. In cases of suspected bilobed or multilobed saccular aneurysms, additional angiographic projections such as transorbital oblique, Caldwell oblique, and oblique-submentovertex should be employed [11]. 3D CTA or 3D rotational angiography aids in this differentiation [14]. However, this distinction may not always be feasible preoperatively, and kissing aneurysms are often discovered intraoperatively.

While conventional angiography remains the gold standard for diagnosing intracranial aneurysms, 3D rotational angiography provides more detailed information about vascular anomalies associated with ACoA aneurysms. Such anomalies can complicate surgical or EVT and include unilateral hypoplasia of the ACA, arterial duplications, fenestrations, dimples, fusion, median artery of the corpus callosum, and azygous ACA [14].

In clinical settings, 3D digital subtraction angiography (DSA) has demonstrated greater sensitivity compared to 2D DSA for detecting small aneurysms and aneurysm remnants [15]. Consequently, 3D DSA is being proposed as the new standard for interventional cerebral vascular imaging due to its superior spatial resolution, 3D imaging, and dynamic information [16]. However, 3D DSA with single-volume reconstruction can be compromised by device-related artifacts, making it challenging to detect recanalization and plan further interventions [17]. To mitigate this, the dual-volume reconstruction technique was developed. This method utilizes three data sets from 3D DSA, namely, native (mask), fill, and subtracted-fill. The mask and subtracted-fill data sets are optimized separately by adjusting contrast, windowing, and color coding, and then fused into a single dual-volume image. These images are especially useful for differentiating vessels from medical devices and understanding their relationship with bone structures, thus enhancing the accuracy in identifying residual lesions [18].

The treatment of ACoA aneurysms poses significant surgical challenges due to their bilateral anterograde arterial supply, deep midline location, and close association with 11 critical arteries and their perforators. These include the paired A1 segments, paired A2 segments, two recurrent arteries of Heubner, two orbitofrontal arteries, two frontopolar arteries, and the ACoA itself [19].

Surgical clipping of kissing ACoA aneurysms presents multiple difficulties. One controversy is the determination of which aneurysm to clip first in the presence of multiple aneurysms [11]. The usual practice is to clip the ruptured aneurysm first, but identifying the ruptured aneurysm intraoperatively can be challenging due to dense clots and adhesions around the rupture and kissing points. Additionally, placing a clip on the first aneurysm may hinder access to the second. An alternative approach is to clip the farthest unruptured aneurysm first, but this can cause traction on the ruptured aneurysm, potentially leading to premature secondary rupture. Kinking of the vessel post-clipping is another concern, and care must be taken to align the clips parallel to the parent vessel. The limited space around each aneurysm neck further complicates clip application [7].

Before placing the first clip, proper preparation of both aneurysms’ necks is essential. Baldawa et al. reported clipping the proximal ruptured aneurysm first, with coagulation and excision of the aneurysmal sac aiding in better visualization of the second unruptured aneurysm [19]. However, the clipping sequence should be adapted to each patient’s unique anatomy.

The presurgical selection of the ideal aneurysm clip in the study involves the use of a 3D planning system to select the appropriate clip geometry for complete aneurysm occlusion. This preoperative planning is critical in determining the most effective aneurysm clip based on morphological characteristics such as size, shape, and the neck of the aneurysm. Schwandt et al. leveraged modern 3D image-processing techniques to simulate different clipping scenarios before the actual surgery. By uploading routine imaging data from patients to a 3D planning workstation, surgeons can assess and select the ideal clip geometry that promises the best fit and occlusion outcome. This preparation is crucial as it allows the surgical team to preselect a specific aneurysm clip based on the morphological characteristics of the aneurysm, such as its diameter, neck width, and dome-to-neck ratio. The morphologic characteristics of an aneurysm significantly influence the presurgical planning process. The size and shape of an aneurysm are pivotal in determining the type of clip needed. For instance, larger aneurysms or those with irregular shapes might necessitate more robust or specially designed clips to achieve complete occlusion. The width and specific morphology of the aneurysm’s neck are also critical considerations. The selected clips need to be precisely positioned to fully seal the neck, which prevents blood from entering the aneurysm sac and subsequently reduces the risk of rupture or regrowth. Additionally, the aneurysm’s location within the cranial space plays a vital role in clip selection and the surgical approach. Aneurysms located in more complex or deeper areas of the brain may require clips that are adaptable to narrower or more angled applications, influencing both the technique and the outcome of the surgical procedure [20].

The 3D planning and presurgical selection of clips play a critical role in significantly mitigating intraoperative difficulties. Detailed presurgical planning minimizes the necessity for intraoperative adjustments of the clips. Surgeons enter the operation with a well-defined plan concerning which clips will be used and their precise application method. Additionally, by selecting the ideal clip and meticulously planning the surgical approach beforehand, surgeons can perform procedures more efficiently and confidently. This preparation not only shortens the duration of the surgery but also reduces the likelihood of potential complications, such as intraoperative aneurysm rupture or incomplete occlusion, thus enhancing both the safety and effectiveness of the procedure.

Given these surgical complexities, endovascular coiling presents as an alternative treatment option for kissing aneurysms, especially when the aneurysms have narrow necks and favorable configurations and the patient is at high risk for general anesthesia [7,21]. Various studies have discussed different cases in the field, highlighting the diverse aspects characterizing the treatment of kissing ACoA aneurysms, as summarized in Table 1.

**Table 1:** Studies about “kissing aneurysms” of the anterior communicating artery (ACoA) and their aspects. N = number of patients; M = male; F = female; NA = not available

First author and year	N	Gender		Maximum diameter (mm)	Hunt and Hess score	Fisher score	Ruptured	Treatment	Postoperative outcomes
		M	F						
Wanifuchi et al 2001 [22]	1	1 (100%)	0 (0%)	NA	NA	NA	One	Clipping	Good recovery
Matsumoto et al 2005 [10]	1	1 (100%)	0 (0%)	3 mm and 4 mm	IV	NA	One	Coil embolization	Good recovery
Inci et al 2005 [11]	6	4 (66.66%)	2 (33.33%)	12 mm and 5 mm; 6 mm and 3 mm; 7 mm and 6 mm; 7 mm and 6 mm; 4 mm and 3 mm; 4 mm and 3 mm	II II IV III III II	NA	One: 3 patients (50%). NA: 3 patients (50%)	All lesions clipped	Good recovery: 3 patients (50%). Moderate disability: 1 patient (16.66%). Deaths: 2 patients (33.33%)
Suh et al 2008 [7]	1	1 (100%)	0 (0%)	2.1 mm and 5.0 mm	NA	3	One	Coil embolization	Good recovery
Baldawa et al 2011 [19]	1	1 (100%)	0 (0%)	NA	NA	NA	One	Clipping	Good recovery

According to the information presented in Table 1, both surgical clipping and endovascular coil embolization have shown favorable outcomes in patients with kissing ACoA aneurysms. These patients typically presented with subarachnoid hemorrhage originating from the kissing aneurysms, with a majority achieving good postoperative recovery.

However, in certain cases, patients also had associated vascular anomalies. For instance, Matsumoto et al. [10] reported a distal ACA aneurysm, while Suh et al. [7] identified a left internal carotid artery bifurcation aneurysm. Due to their small size, these aneurysms were not amenable to embolization and were instead treated through surgical clipping. Consequently, in these instances of coil embolization for kissing aneurysms, surgical clipping was also employed as part of the treatment strategy.

EVT of kissing aneurysms offers certain advantages over surgical neck clipping. It reduces risks associated with open surgery, such as premature rupture during dissection and brain injury during retraction to create an adequate operative field. EVT is particularly effective in protecting against premature rupture and brain injury. Moreover, with EVT, the decision about the treatment order becomes less critical, as the untreated aneurysm is not subject to surgical retraction. Coil embolization is thus highly beneficial for kissing aneurysms closely originating from the same parent artery, such as those at the internal carotid-posterior communicating artery, internal carotid-anterior choroidal artery, or ACoA locations [23].

While EVT of kissing aneurysms offers certain advantages, it also has limitations. One significant challenge is the difficulty in immediately controlling bleeding if an aneurysm on the opposite side ruptures during coiling [10]. Additionally, some intracranial kissing aneurysms, such as those in the distal ACA, may present with small domes or broad necks, making them less suitable for coil embolization.

Advancements in endovascular interventional techniques and materials have expanded the range of intracranial aneurysms treatable by endovascular methods, including the use of flow diverters. However, the complex structure of ACoA aneurysms can still pose challenges for EVT. Issues such as poor aspect ratio (dome/neck), difficulty in catheter placement, and the potential impact on blood flow in collateral vessels can make EVT unfeasible. Moon et al. reviewed the long-term prognosis of ACoA aneurysms included in the Barrow Ruptured Aneurysm Trial (BRAT). In this study, 91 (70%) patients with ACoA aneurysms were assigned to the clipping group, while 39 (30%) patients were in the embolization group [24]. In the embolization group, 16.9% of the patients crossed over to the surgical clipping group due to difficulties with embolization. The clinical outcomes between the two groups showed no significant difference after one to three years of follow-up. However, the retreatment rate was lower in the surgical clipping group compared to the embolization group, with 3.3% of patients in the clipping group and 7.7% in the coiling embolization group requiring retreatment. Notably, no patients were transferred from surgical clipping to coiling embolization.

According to the Cerebral Aneurysm Rerupture After Treatment (CARAT) study, the degree of aneurysm occlusion post-treatment plays a pivotal role in predicting the risk of re-hemorrhage in patients with treated ruptured intracranial aneurysms. The study highlighted that complete occlusion of an aneurysm correlates with the lowest risk of re-rupture, noting a significant escalation in risk with less comprehensive occlusion levels. Specifically, re-rupture rates were 1.1% for complete occlusion, rising to 2.9% for 91% to 99% occlusion, 5.9% for 70% to 90% occlusion, and 17.6% for less than 70% occlusion. The findings also revealed that most re-ruptures occurred shortly after the treatment, with a median time to re-rupture of only three days, and the risk diminished substantially after the first year [25].

The CARAT study also compared the effects of treatment methods, where coil embolization was associated with a slightly higher re-rupture risk compared to surgical clipping. However, this difference was not statistically significant after adjusting for occlusion degree and other variables. Achieving complete occlusion is emphasized as essential for minimizing re-rupture risk. Patients not achieving complete occlusion require vigilant monitoring and possibly early re-treatment to manage the elevated risk effectively. Furthermore, the study supports consistent follow-up imaging for all patients, especially within the first year of treatment, to promptly identify and manage potential re-ruptures. This approach is crucial given that the risk of re-rupture, although lower with complete occlusion, remains non-negligible [25].

## Conclusions

To summarize, intracranial “kissing” aneurysms represent a significant challenge in the field of vascular neurosurgery, particularly with aneurysms located at the ACoA. EVT can be complicated by the intricate structure of ACoA aneurysms and the associated difficulties in controlling bleeding during the procedure. In contrast, surgical clipping often provides a more direct and effective approach, with some cases necessitating surgical intervention even after initial endovascular efforts.

Current literature might suggest that surgical clipping results in a reduced need for subsequent interventions compared to coiling embolization, although comprehensive elaboration on this comparison is somewhat limited. The successful surgical treatment and subsequent recovery of our patient further contribute to the body of knowledge surrounding the management of this complex disease, underscoring the importance and effectiveness of tailored surgical approaches in addressing these formidable vascular pathologies.
